# Progress in injectable hydrogels for the treatment of incompressible bleeding: an update

**DOI:** 10.3389/fbioe.2023.1335211

**Published:** 2024-01-09

**Authors:** Xiudan Wang, Xinran Yang, Zhiguang Sun, Xiaoqin Guo, Yanjiao Teng, Shike Hou, Jie Shi, Qi Lv

**Affiliations:** ^1^ Institution of Disaster and Emergency Medicine, Tianjin University, Tianjin, China; ^2^ Wenzhou Safety (Emergency) Institute of Tianjin University, Wenzhou, China; ^3^ Key Laboratory for Disaster Medicine Technology, Tianjin, China

**Keywords:** injectable, hydrogel, noncompressible haemostasis, haemostatic material, biomaterial

## Abstract

Uncontrollable haemorrhage from deep, noncompressible wounds remains a persistent and intractable challenge, accounting for a very high proportion of deaths in both war and disaster situations. Recently, injectable hydrogels have been increasingly studied as potential haemostatic materials, highlighting their enormous potential for the management of noncompressible haemorrhages. In this review, we summarize haemostatic mechanisms, commonly used clinical haemostatic methods, and the research progress on injectable haemostatic hydrogels. We emphasize the current status of injectable hydrogels as haemostatic materials, including their physical and chemical properties, design strategy, haemostatic mechanisms, and application in various types of wounds. We discuss the advantages and disadvantages of injectable hydrogels as haemostatic materials, as well as the opportunities and challenges involved. Finally, we propose cutting-edge research avenues to address these challenges and opportunities, including the combination of injectable hydrogels with advanced materials and innovative strategies to increase their biocompatibility and tune their degradation profile. Surface modifications for promoting cell adhesion and proliferation, as well as the delivery of growth factors or other biologics for optimal wound healing, are also suggested. We believe that this paper will inform researchers about the current status of the use of injectable haemostatic hydrogels for noncompressible haemorrhage and spark new ideas for those striving to propel this field forward.

## Graphical Abstract

**Figure:**
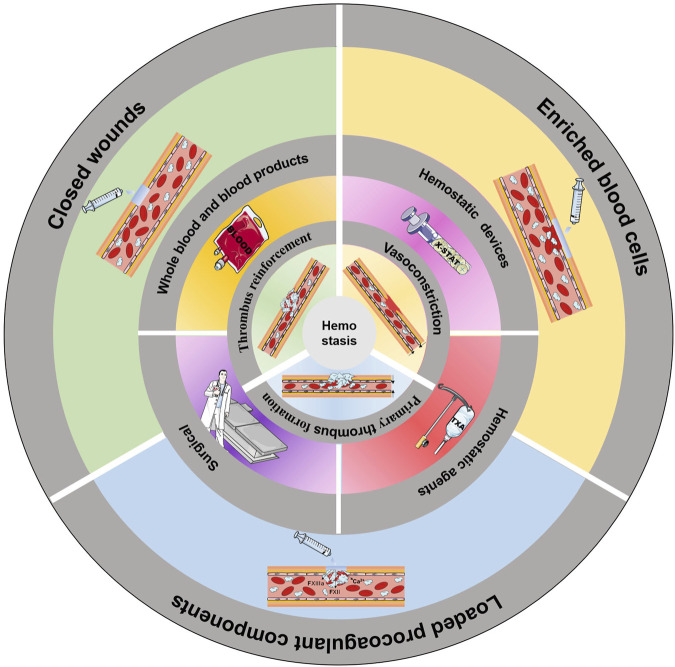
Common methods of haemostasis. The innermost circle shows the three stages of haemostasis. The middle circle shows the common clinical methods to induce haemostasis. The outermost circle illustrates the use of an injectable hydrogel to induce haemostasis.

## 1 Introduction

Traumatic bleeding remains a significant cause of death, resulting in more than 1.5 million deaths each year (Shi et al., 2021). The type of haemorrhage that accounts for the highest percentage of deaths is noncompressible haemorrhage, which is responsible for up to 90% of deaths in war and 40% of deaths in civilian life (Jamal et al., 2021). Therefore, rapid and effective haemostasis, especially for noncompressible haemorrhage, is crucial for saving lives (Guo et al., 2021; Peng et al., 2021). With gradually increased understanding of the haemostatic process, various haemostatic materials that mimic the human haemostatic mechanism have been developed. Tourniquets, gauze, sponges, and bioadhesives are common types of haemostatic materials, and some marketed haemostatic materials, such as X-STAT, junctional tourniquets and pelvic binders, have shown excellent haemostatic properties in surgical and battlefield emergencies (Zhang et al., 2021). However, there is still a lack of effective treatments for noncompressible bleeding in the chest and abdominal organs.

Hydrogels have a three-dimensional porous structure, exhibit good hydrophilicity, are easily modified, and can be designed to be injectable. Moreover, these materials have great potential to solve the problems of the inability to cover the wound completely, reach the bleeding point, and compress the wound because of their flexible drug delivery, strong tissue adhesion, strong water absorption, blood cell enrichment, and haemostatic component loading (Yu and Ding, 2008; Chin et al., 2019). Through continuous research efforts, the functionality of hydrogels has changed from simple physical coverage or single functionality to multiple properties (Patenaude et al., 2014; Ahmed, 2015; Liu et al., 2015), providing new options for haemostatic treatment. Here, we provide an overview of the coagulation mechanisms and recent applications and research progress of haemostatic materials, especially injectable hydrogels, based on different coagulation mechanisms. We will also discuss the outlook on the challenges and potential in this field.

## 2 Mechanisms of haemostasis and treatment of noncompressible haemorrhage

### 2.1 Mechanisms of haemostasis

**FIGURE 1:**
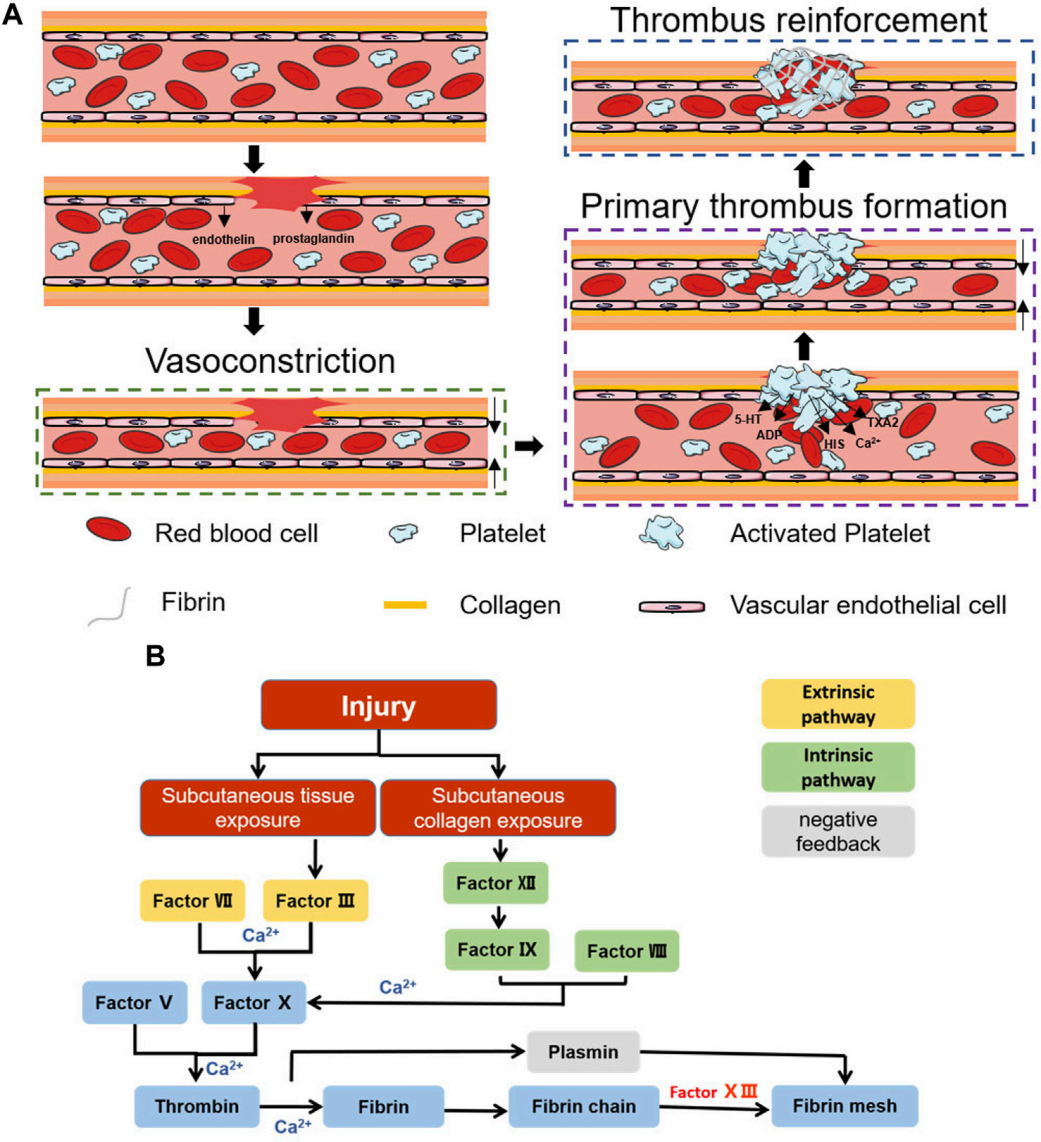
Schematic diagram of the haemostatic process. **(A)** Schematic diagram of the coagulation process. **(B)** Schematic diagram of the coagulation cascade reaction.

Coagulation is a natural physiological process that occurs when the body is traumatized and prevents the continuous flow of blood from broken vessels, mainly through thrombus formation. Haemostasis can be divided into three stages: vasoconstriction, primary thrombosis, and thrombus reinforcement (Figure 1A) (Furie and Furie, 2008).

#### 2.1.1 Vasoconstriction

After vascular injury, a reflexive vascular smooth muscle spasm occurs (Rodrigues et al., 2019), causing rapid vascular contraction after injury, while the damaged vascular endothelium releases endothelin and the damaged cells release prostaglandins, all of which promote vasoconstriction (Godo and Shimokawa, 2017). Vasoconstriction causes a rapid decrease in blood flow and reduces blood loss. However, this stage of haemostasis is transient, and the hypoxic environment of the wound decreases the local pH, causing relaxation of the vascular endothelium, at which blood flow and bleeding resume (Pool, 1977). To achieve prolonged vasomodulation, mediators such as fibrinopeptides, 5-hydroxy tryptamine (5-HT) and thromboxane A2 (TXA2), which are released after activation of the coagulation cascade, are necessary (Strecker-McGraw et al., 2007; Teller and White, 2009).

#### 2.1.2 Primary thrombus formation

This stage is dominated by the formation of platelet thrombi. Platelets were first discovered by Schultze in 1865 as enucleated cells detached from megakaryocytes (Frojmovic and Milton, 1982; Chan Choi et al., 2014). In the presence of intact blood vessels, endothelial cells have antithrombotic properties: they generate nitric oxide (NO), prostacyclin and negatively charged heparin-like glycosaminoglycans, preventing platelet activation, adhesion and aggregation (Golebiewska and Poole, 2015). After vascular injury, collagen exposure can cause platelet adhesion, and integrins on the platelet surface are activated after adhesion occurs. αIIbβ3 is the most abundant integrin on platelets and can promote further platelet aggregation and adhesion by mediating the attachment of fibrinogen, fibronectin, and vascular haemophilia factor (vWF) through RGD sequences (Wagner et al., 1996; Nuyttens et al., 2011). Activated platelets also undergo significant morphological changes, and the filamentous actin content in activated platelets can reach 70%. Moreover, the conformational change in filamentous actin can change platelets from a disk-like structure to an omelette-like structure with multiple pseudopods, which allows platelets to bind tightly to the extracellular matrix (ECM), constrict blood vessels and seal wounds (Sorrentino et al., 2015). Moreover, these activated platelets accelerate platelet activation and aggregation by secreting ADP, 5-HT, calcium (Ca^2+^), histamine (HIS), TXA2, and more than three hundred other active substances (Blair and Flaumenhaft, 2009; Golebiewska and Poole, 2015). Under the combined effect of the above pathways, the initial formation of platelet plugs seals the wound and stops bleeding.

#### 2.1.3 Thrombus reinforcement

Primary thrombus formation temporarily closes the wound, stopping the bleeding. However, at this time, the thrombus is not stable, and rebleeding is likely to occur due to external forces. Further reinforcement is necessary, a step we often refer to as the coagulation cascade reaction. The coagulation cascade reaction can be divided into two pathways: endogenous and exogenous (Gale, 2011). Both pathways promote the formation of the fibrin network by inducing prothrombogenic fibrin to form thrombin via coagulation factor X (FX) (Versteeg et al., 2013). However, in the exogenous haemostatic pathway, subcutaneous tissues activate FC via factor VII and factor III (prothrombin complex) (Nemerson, 1988; Mackman, 2009; Tormoen et al., 2013). In contrast, in the endogenous haemostatic pathway, subcutaneous collagen activates FX via factor XII, factor VIII, and factor IX (Bhopale and Nanda, 2003; Tormoen et al., 2013) (Figure 1B). The cascading amplification effect of the coagulation cascade reaction and the anionic surface of the platelet peg contribute to the rapid adhesion of blood cells and rapid fibrin network formation, which together complete the thrombus reinforcement process (Morrissey et al., 2012). Correspondingly, the negative feedback regulatory pathway represented by fibrolase maintains the overall dynamic balance and prevents excessive thrombus formation (Hoylaerts et al., 1982; Travis and Salvesen, 1983). The completion of thrombus reinforcement marks the end of the haemostatic process.

### 2.2 Treatment of noncompressible haemorrhage

**FIGURE 2:**
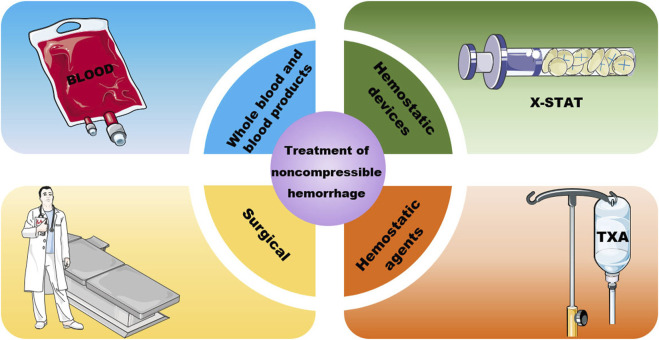
Treatment of noncompressible haemorrhage.

Currently, there are three clinical treatments for noncompressible trunk haemorrhage: blood transfusion, haemostatic devices and materials, and surgical or endovascular measures (Figure 2). All of these methods have their own advantages and disadvantages and can be chosen on a case-by-case basis.

#### 2.2.1 Whole blood and blood products

To date, whole-blood (WB) transfusions remain the most commonly used clinical treatment for noncompressible haemorrhage (Cap et al., 2018). The Committee on Tactical Combat Casualty Care (CoTCCC) gives resuscitation sequence recommendations for treating haemorrhagic shock, as shown in Table 1 (Butler et al., 2014).

**TABLE 1:** Resuscitation sequence recommendations for treating haemorrhagic shock.

NO.	Type of resuscitation fluid
1	Whole blood
2	1:1:1 plasma: RBC: platelet
3	1:1 plasma: RBC
4	Plasma or RBC
5	Hydroxyethyl starch
6	Ringer’s lactate or plasma-lyte A

However, the storage requirements for WB are very high, and it is difficult to obtain WB in real time on battlefields and at disaster sites; thus, freeze-dried plasma attracted much attention during the Second World War. Freeze-dried plasma is a solid powder of WB obtained after virus inactivation and freeze-drying. The stability and shelf life of blood after freeze-drying are greatly increased, and freeze-dried plasma is widely used in prehospital treatment (Kendrick douglas et al., 1965).

#### 2.2.2 Haemostatic devices and agents

Although blood transfusions are effective in the treatment of noncompressible haemorrhage, blood products are available from limited sources and have high storage costs and limited shelf life. To meet clinical needs, various haemostatic devices and agents have been developed. A variety of products have been marketed, each with its own advantages, and have solved some clinical problems; however, there are still limitations that need to be further addressed. Several of these devices and agents are compared in Table 2.

**TABLE 2:** Haemostatic devices and agents.

Name	Mechanism	Advantages	Disadvantages	References
Abdominal aortic and junctional tourniquet (AAJT)	Pressure on the abdominal aorta or junction after inflation of the balloon	Compression strength can be controlled by inflation volume	Pain, inflammation, liver or intestinal ischaemia	Anonymous (2013), Croushorn et al. (2013), Croushorn (2014), Brännström et al. (2018)
Pelvic binder	Reduce the pelvic volume and increase the pressure in the pelvic cavity	Can be used for haemostatic treatment of unspecified bleeding points	Early trauma does not stop bleeding	Ghaemmaghami et al. (2007), Montgomery et al. (2017), Morris et al. (2017)
iTClamp	Works like a clamp, closes the wound	Simple to apply and less painful	Can only be applied to superficial wounds	Filips et al. (2013), St John et al. (2015), Onifer et al. (2019), Stuart et al. (2019)
XStat	Quickly concentrates blood to promote haemostasis, swells to compress the wound	Can be used for penetrating, rapid haemostasis	Cannot be left in the wound for a long period of time but is difficult to remove from the wound	Sims et al. (2016), Cox and Rall (2017), Rodriguez et al. (2017), Stuart et al. (2019), Warriner et al. (2019), Bonanno et al. (2020)
ResQFoam	Foam expands up to 30 times and becomes solid in contact with liquid, allowing compression of the wound	Easy to operate, can be used by nonprofessionals, less traumatic	Poor for arterial haemorrhage, leads to abdominal compartment syndrome, bowel injuries, thermal injuries	Chang et al. (2015)
Antifibrinolytic tranexamic acid (TXA)	Antifibrinolytic properties	Administered intravenously, effective for most haemorrhages	Must be used within 3 h of injury	Roberts et al. (2011), Zhang (2018), Picetti et al. (2019), Hanley et al. (2021)

#### 2.2.3 Surgery

Open surgery and endovascular measures become necessary when other treatment methods fail to stop bleeding. Open surgery includes packing, vascular ligation, the removal of solid organs, nonanatomical organ resection and temporary shunting, and it requires not only specialized surgeons but also a fully equipped and sterilized operating room, which is difficult to achieve in a hostile environment. A higher risk of infection and longer recovery time can also occur. As endovascular surgical techniques continue to develop, resuscitative endovascular balloon occlusion of the aorta (REBOA) is being used in a variety of haemostatic scenarios. REBOA can increase vital organ perfusion as well as cardiac afterload and aortic pressure by blocking distal blood flow and balloon bleeding. REBOA significantly improves survival and reduces complications compared to open surgery (Taylor et al., 2017). After insertion and expansion of the sacculus, various imaging methods, such as fluoroscopy, ultrasound, and radiography, can be used to determine whether the designated placement site has been reached. Physicians can also perform blind insertion in emergencies or in prehospital care. Successful placement is determined by changes in systolic blood pressure.

Overall, blood products are still the first choice for clinical haemostasis, but they do not fully meet clinical needs due to factors such as source limitations, preservation, and the risk of viral transmission. Advances in haemostatic devices and agents have increased interest in haemostatic treatment, but these devices can only meet the needs of emergency haemostasis or auxiliary haemostasis and cannot completely replace surgical treatment. Moreover, there are strict requirements for the injury site and the degree of injury. Surgical treatment is the most reliable solution for haemostasis, but it is not suitable for prehospital rescue because of the high requirements for the environment and operators.

## 3 Injectable hydrogels

### 3.1 Advantages of injectable hydrogels

Hydrogel materials have received much attention in recent years; they are three-dimensional polymer networks obtained by crosslinking through chemical bonds or physical interactions that can absorb large amounts of water while maintaining their structural integrity (McKinnon et al., 2014). The three-dimensional network structure resembles the ECM structure and has good biocompatibility while maintaining satisfactory permeability and facilitating the release of loaded components (Schmidt et al., 2008); this structure also endows the hydrogel with good water absorption properties, which are beneficial for haemostasis (Liu et al., 2015). A high degree of hydration facilitates the loading and rapid release of water-soluble components (Hoare and Kohane, 2008; Li and Mooney, 2016), facilitating the incorporation of water-soluble haemostatic components such as tranexamic acid and thrombin (Ker et al., 2012; Morrison et al., 2012; Tripodi, 2016). More importantly, hydrogels can be endowed with additional functions by modifying the polymers that constitute the hydrogel backbone, such as the addition of catechol moieties, to increase the viscosity of hydrogels (Guvendiren et al., 2012). The multiple advantages of hydrogels have made them a topic of intense interest in haemostatic materials (Ahmed, 2015). In recent years, injectable hydrogels have acquired a broader range of applications (Yu and Ding, 2008): injectable hydrogels can be used to solve the problem of deep wound haemostasis facilitating minimally invasive surgery. The fluid precursor of *in situ* gel-forming injectable hydrogels can be used to achieve complete coverage of irregular wounds and precise delivery to the bleeding location with the help of professional delivery devices (Patenaude et al., 2014), addressing deep wounds and noncompressible bleeding.

### 3.2 Injectable hydrogel design strategy

The design of injectable hydrogels involves two main steps: selection of the backbone material and the crosslinking method. The density of hydrophilic groups on the backbone material determines the water absorption capacity, and adequate water absorption is critical for haemostatic materials. Excellent backbone polymers can absorb up to several thousand times their own weight in water (Bhujbal et al., 2020). However, swelling after water absorption will cause rapid disintegration of the material or inability to form gels, so crosslinking is needed to promote the transition from sol to hydrogel or to improve the mechanical properties of the hydrogel. Currently, the commonly used crosslinking methods include physical crosslinking and chemical crosslinking (Lee, 2018; Zhu et al., 2019; Bashir et al., 2020; Zawani and Fauzi, 2021) (Table 3).

**TABLE 3:** Injectable hydrogel design strategy.

Crosslinking method	Mechanism	Advantages	Disadvantages
Physical	Electrostatic interactions	High biosafety, no crosslinking agent required	Reversible crosslinking, low increase in on mechanical strength
	Hydrophobic interactions		
	Host-guest interactions		
	Van der Waals forces		
Chemical	Diel–Alder	High crosslinking efficiency, crosslinking stability, can significantly increase mechanical strength	Some crosslinkers pose biosafety risks and have long degradation cycles *in vivo*
	Michael addition		
	Enzyme-mediation		
	Photopolymerization		

In addition, a number of other factors, such as temperature, pH, electric field, magnetic field, light, and enzymes, can also affect the crosslinking and state of injectable hydrogels. These characteristics have been exploited to develop “smart” biomaterials. For example, injectable haemostatic materials that respond to temperature changes have been designed (Ryu et al., 2011).

### 3.3 Design requirements for injectable haemostatic hydrogels

Rapid haemostasis is the most important requirement for a haemostatic material. Injectable hydrogels with adhesive sealing properties are ideal for bleeding sites that cannot be directly compressed (Han et al., 2020). However, such sites present great challenges to material performance. In addition to the moist environment in the case of bleeding, the effects of blood pressure in the arteries also impose high requirements on the adhesive and burst strength of the material (Fan et al., 2020; Kim et al., 2020; Baghdasarian et al., 2022). For materials that achieve haemostasis by enriching blood cells, sites for easy cell loading or strong fluid absorption are key to material design. For materials that activate the coagulation cascade, the appropriate choice of components is critical to successful design.

In addition, to address noncompressible bleeding from in organs and blood vessels in the torso, high biosafety of the material is also essential. In addition to meeting regulatory requirements, the inability to cause excessive thrombosis is a key concern in the development of haemostatic hydrogels.

Finally, timely and rapid intervention with a short therapeutic invasive period is needed for patients with uncontrollable bleeding. Haemostatic hydrogels should be portable, simple to use, and operable not only by medical personnel but also by civilians without a medical background. To be useful in prehospital emergency scenarios, the material should not have strict requirements for storage conditions or allow too short a storage time.

## 4 Injectable hydrogels for the treatment of noncompressible trunk haemorrhages

**FIGURE 3:**
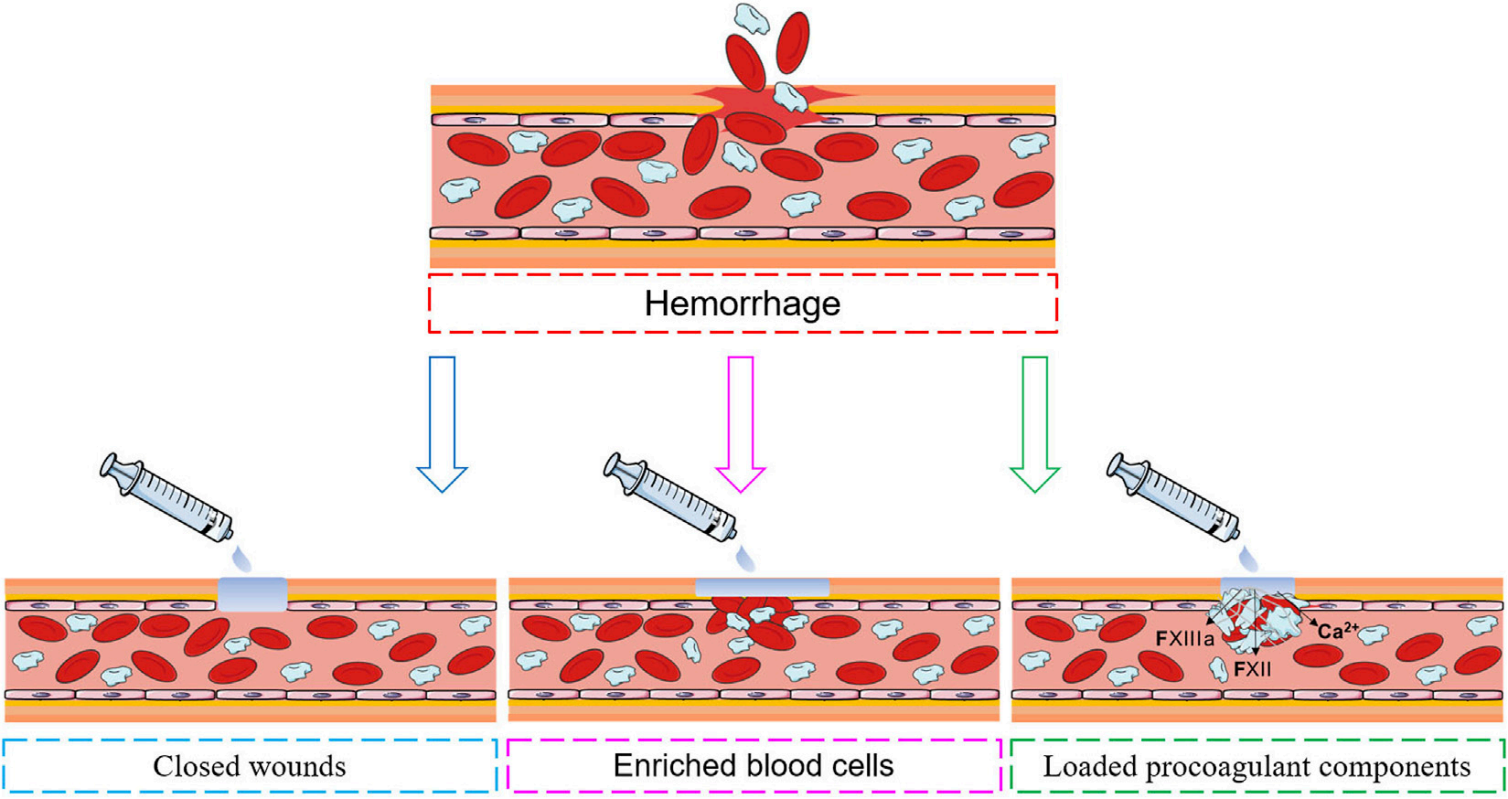
Injectable haemostatic hydrogel haemostatic mechanisms.

With increasing understanding of the haemostatic process, a variety of haemostatic materials that mimic the human haemostatic mechanism have been developed (Patenaude et al., 2014). The haemostatic mechanisms used can be divided into three categories: wound closure, blood cell enrichment, and the loading of procoagulant components (Figure 3).

### 4.1 Closed wounds

**FIGURE 4:**
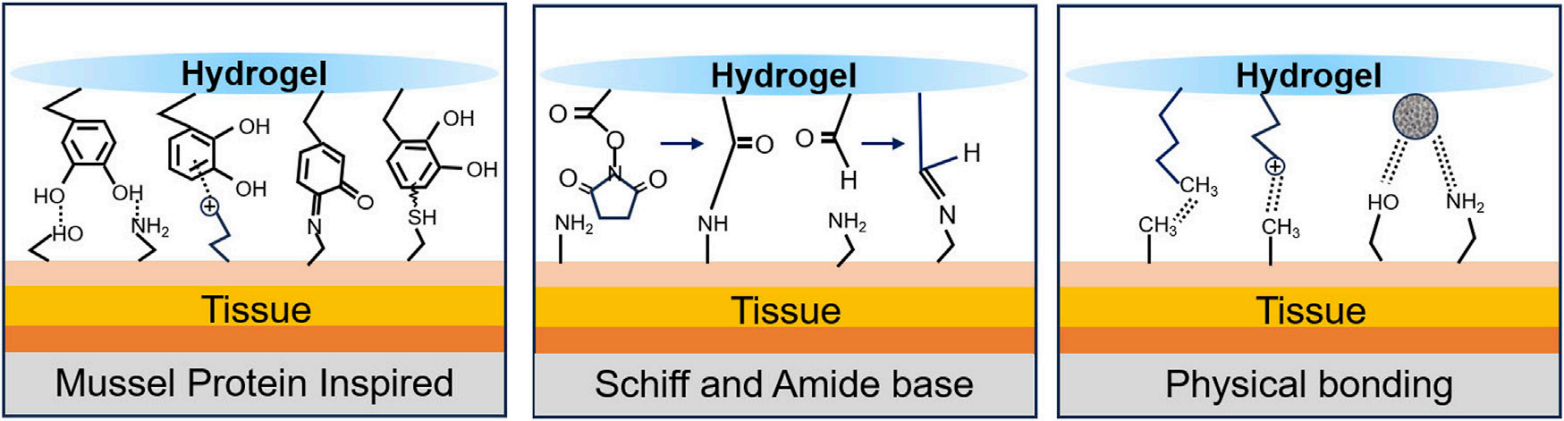
Schematic diagram of the bonding principle.

Closing or minimizing the wound is the most direct and effective way to stop noncompressible bleeding, similar to early vasoconstriction or complete thrombosis in the early stages of bleeding. A well-bonded hydrogel can quickly and effectively stop further blood flow (Zhang et al., 2020). Figure 4 shows the common principles of hydrogel wound closure. Table 4 summarizes the adhesive strength of representative hydrogel adhesives.

**TABLE 4:** Representative research on adhesion strength and adhesion composition/structure.

Material	System	Maximum adhesion strength (kPa)	Adhesive composition/structure
HA (CHO)-QCS (DOPA)	Aldehyde-functionalized hyaluroni, acid–catechol-functionalized quaternized chitosan	140	Catechol, aldehyde group
iCMBAs	PEG, dopamine, citric acid and sodium periodate	123.0	Catechol
PPD	Poly (lysine) polyethylene glycol backbone, HRP	147	Catechol, polylysine
SFP	TA, silk protein	130.0	Polyphenol, SF
RAAS	Methacrylated hyaluronic acid, PF127, AA-NHS	33.0	NHS
CQCS@gel	CQCS, DB-PEG 2000	33.5	Catechol, aldehyde group
GMDA	Dopamine-modified methacrylate gelatine, peroxidase, hydrogen peroxide	134.3	Catechol
CGD	Chitosan and DHCA, β-glycerophosphate	32.5	Catechol
PEG-LZM	4-arm-PEG-NHS, sodium tetraborate, lysozyme	20	NHS
St-Dopa	Dopamine, carboxylic starch, HRP, hydrogen peroxide	95.5	Catechol
CMCS-brZnO	CMCS, brZnO	45.0	Electrostatic interaction, hydrogen bonding
AHES/ACC	AHES, ACC	42.7	Aldehyde group, electrostatic
PDPC	PVA, DOPA, Cu^2+^	26.0	Catechol
DNGel	Catechol-Fe^3+^, NIPAAm-methacryloyl	3,500.0	Catechol
DGM	Amylopectin from maize, gelatine, MBGN	107.6	Hydrogen bonding, electrostatic interaction
PBO	PVA, borax, OPC	29.2	Catechol, hydroxy
CSO	CMCS, SA, ODE	120.6	Aldehydes, amino groups, carboxyl groups
GTB	Gelatine, TA, borax	68.0	Polyphenols, Amino
CD	CtNWs, CMCS, DDA	33.2	Hydrogen bonding, electrostatic interaction
PPBA-PVA	poly (N,N-dimethylethylenediaminephazene), PVA, polymer solution modified with phenylboronic group	45.0	Cation–π, π–π, hydrogen bonding

#### 4.1.1 Mussel protein inspiration

The discovery that mussel proteins exhibit strong adhesion underwater has inspired researchers. 3,4-Dihydroxyphenylalanine (DOPA), which has a catechol structure, is widely considered to be the key to the wet adhesion of mussel proteins (Strausberg and Link, 1990; Lee et al., 2006). This is mainly due to the ease with which the catechol moiety can be oxidized chemically or enzymatically to form reactive quinones. These species can further undergo Michael addition and Schiff base reactions with tissue surface nucleophilic reagents such as amines, thiols and imidazole. These reactions can also synergize with hydrogen bonding, π-π stacking, and cation-π interactions to achieve desirable tissue adhesion through multiple pathways (Strausberg and Link, 1990; Lee et al., 2006; Wang et al., 2017; Chen et al., 2018; Xie et al., 2020).

Adding a catechol structure to the material system can effectively improve the tissue adhesion properties. The most common methods involve the direct incorporation of dopamine or tannic acid (TA) into the material body or functional modification of the backbone structure using dopamine. Mehdizadeh et al. (2012) prepared a hydrogel by mixing PEG, dopamine, citric acid and sodium periodate. Its tissue adhesion strength reached 123 kPa (Mehdizadeh et al., 2012). In the work of Jae Park, TA was introduced into a double network hydrogel consisting of poly (vinyl alcohol) (PVA) and poly (acrylic acid) (PAA) to create tissue adhesives (shear strength of ≈31 kPa) and haemostatic agents (bleeding reduced by more than 80%) (Park et al., 2023). Wei et al. (2023) prepared aldehyde-functionalized hyaluronic acid-catechol-functionalized quaternized chitosan [HA (CHO)-QCS (DOPA)] by grafting the catechol structure onto chitosan. The material offers excellent adhesion strength (Wei et al., 2023). This solution can be further used in combination with other functionalized chitosans to enrich the functionality of the material. For example, preparing CQCS@gel with quaternized chitosan as the main raw material increases the antimicrobial properties of the material (Fang et al., 2023), and HBCS-C prepared using hydroxy butyl chitosan has excellent temperature sensitivity (Shou et al., 2020). In addition to chitosan, hyaluronic acid (HA), sodium alginate (SA), and gelatine can exhibit increased viscosity through dopamine modification (Ryu et al., 2011; Chen et al., 2018; Liang et al., 2019a; Song et al., 2019; Wang et al., 2020; Xia et al., 2022; Yang et al., 2022). The HACN network designed by Xia et al. (2022) is representative. It consists of thiourea-conjugated HA (HA-NCSN) and catechol-conjugated HA (HA-Cat). HACN has been demonstrated to have a good haemostatic effect on the digestive tract in *in vivo* experiments in pigs (Xia et al., 2022). By modifying collagen and starch chains with the structure of catechols in mussel proteins and the structure of arabinogalactan proteins in creeping plants, the proteins can then be crosslinked by calcium ions to form the hydrogel CoSt. CoSt shows repeatable strong wet tissue adhesiveness (62 ± 4.8 KPa), high sealing performance (153.2 ± 35.1 mmHg), and high haemostatic efficiency compared with fibrin glue (Yang et al., 2023).

Previous studies have confirmed that adhesiveness is determined by the balance and synergy of interfacial adhesion and cohesion qualities inside the sticky matrix (Li et al., 2017; Bu and Pandit, 2022). The introduction of enzymes [e.g., horseradish peroxidase (HRP)] into the material system not only increases the crosslinking strength of the material and thus the cohesion but also aids in the conversion of catechol to catechol aldehyde, which further increases the adhesive properties of the material. Guoqing Wang synthesized highly branched gelatine containing a large number of catechol terminals via synergetic crosslinking of catechol-Fe^3+^ chelation and HRP/H_2_O_2_-triggered covalent bonds, yielding shear strength of 115.0 ± 13.1 kPa and sealing strength of 245.0 ± 33.8 mmHg (Wang GQ. et al., 2022). In the hydrogel prepared by Wang et al. (2017), the catechol structure was grafted onto the poly (lysine) polyethylene glycol (PEG) backbone, catalysed by HRP. The interaction between the catechol structure and lysine resulted in excellent adhesion to dry or wet pigskin, with adhesion strengths up to 147 kPa. The haemostatic effect in a rat liver injury model was also significantly better than that of the fibrin glue group (Wang et al., 2017). Dopamine-coupled starch (St Dopa) macromolecular monomers, which function with the assistance of HRP and hydrogen peroxide to increase the speed of crosslinking and the strength of adhesion, can be injected into the wound surface to quickly form a tough protective barrier to prevent blood loss (Cui et al., 2020). Zhou et al. (2022) utilized GMDA as the main component and introduced photocrosslinking in addition to enzymatic crosslinking to form a dual network structure, which makes the material highly adhesive and able to withstand blood pressures up to 250 mmHg. It has good development prospects in the field of arterial haemostasis (Shin et al., 2015; Bai et al., 2019; Zhou et al., 2022; He et al., 2022; Kang et al., 2023; Liu et al., 2023). Notably, the complexation of catechol with metals can also effectively increase the crosslinking strength of the material and improve its adhesion properties (Wang et al., 2020; Hou et al., 2022). Hu et al. (2023) prepared PVA-DOPA-Cu (PDPC) by esterification between PVA and DOPA and catechol coordination between Cu^2+^ and the catechol moiety of DOPA. Pig liver, heart, and carotid artery haemorrhage experiments were able to stop bleeding rapidly, significantly improving survival rates.

In addition to increasing the grafting rate of catechol, increasing the exposure of the catechol structure can also result in superior tissue adhesion. Cui et al. (2019) attached the catechol structure to the hydrophobic backbone and subsequently contracted the structure in the liquid environment to expose the catechol structure and achieve high adhesion performance.

#### 4.1.2 Schiff and amide bases

Based on the presence of multiple amino structures in tissues, researchers have increased tissue adhesion by preparing materials containing aldehyde structures or NHS for Schiff base or amide reactions with tissues (Xie et al., 2022). Liu et al. (2020) prepared a series of AHES/ACC hydrogels formed from aldehyde hydroxyethyl starch (AHES) and amino carboxymethyl chitosan (ACC). It was also found that varying the ratio of aldehyde to amino groups could tune the properties of the AHES/ACC hydrogels, including gelation time, tissue adhesion strength, swelling rate, degradation and mechanical stretching. Taking advantage of this feature, researchers have prepared Schiff base haemostasis hydrogels with different properties by raw material selection and grafting rate modulation. Cao et al. (2019) constructed a haemostatic hydrogel with carboxymethyl chitosan (CMCS), gelatine and oxidized SA that achieved *in situ* curing within 30 s and reduced bleeding after liver injury in rats by 82.2%. Hao et al. (2022) constructed a haemostatic hydrogel with four-arm-PEG-CHO and polyethyleneimine with a polyamine structure that achieved an *in situ* curing speed of up to 9 s, and the haemostatic speed was comparable to that of Surgiflo^®^. Li et al. (2022) used glycol chitosan and oxidized HA to construct an injectable haemostatic hydrogel with very strong self-healing properties, prolonging the time the material protects the wound. Xie et al. (2022) reported a multifunctional CSO hydrogel prepared from moist pig skin using CMCS, SA, and oxidized dextran (ODex) with a lap shear strength of up to 120.6 kPa. To further optimize the material properties, researchers have combined various design strategies. The effectiveness of modifying the polyphenol structure on the material backbone (Liu et al., 2022), selecting synthetic raw materials with photocrosslinking properties (Chen et al., 2021), and adding nanosubstances to increase the interactions between the raw materials (Zhu et al., 2017; Pang et al., 2020) have been verified by *in vivo* animal experiments. Notably, Hong et al. (2019) used methacrylate gelatine and butyramide-modified HA to construct a dual crosslinked network hydrogel with UV-induced photocrosslinking and a Schiff base reaction. This material successfully achieved haemostasis of the porcine carotid artery and heart because of its high internal systemic strength and high external tissue adhesion strength (Hong et al., 2019). In addition, the oxidized indole moiety on serotonin can react with amine, thiol, and imidazole residues in ECM proteins and carbohydrates via a Schiff base reaction, which enables the hydrogel to adhere firmly to the wound and seal it, achieving haemostasis. Zhang et al. (2021) designed a CSS hydrogel that reduced bleeding in liver-injured rats by 80%.

In recent years, the application of N-hydroxysuccinimide (NHS) has expanded. Like aldehyde groups, NHS can not only increase the crosslinking strength inside hydrogels and regulate the gelation time of materials but also increase the adhesion strength of materials by binding to amino groups on tissue surfaces (He et al., 2021). Numerous experiments have demonstrated that NHS is not involved in the crosslinking of materials but plays a decisive role in the tissue adhesion of materials (Ye et al., 2022). Owing to their tuneable performance and high biosafety, these materials have a wide range of applications. In addition to effectively controlling rat liver and blood vessel haemorrhage, they also exhibited ideal haemostatic effects on rat cerebral cortex injury, cerebrovascular injury, cardiac injury, and porcine gastric haemorrhage (He et al., 2021; Bian et al., 2022). The addition of borax further increased the gel formation speed. The modulation of the ratio between the components can realize rapid modulation of the wound and enable long-distance delivery in conjunction with minimally invasive surgery (Tan et al., 2021).

#### 4.1.3 Physical bonding

In addition to chemical crosslinking, physical bonding affects tissue adhesion. Grafting long-chain alkyl groups on macromolecular backbones has been reported to increase tissue adhesion by interacting with alkyl groups in subcutaneous adipose tissue. Du and Chen used this principle to prepare adhesive hydrogels by mixing long-chain alkyl-modified chitosan with ODex or PEG (Chen et al., 2018; Du et al., 2019a). Positively charged materials (e.g., chitosan and gelatine) can increase adhesion to biological tissues through electrostatic interactions (Hoque et al., 2017). The use of positively charged groups [e.g., ammonium groups, quaternary ammonium groups, and ε-polylysine (ε-PL)] to modify the material backbone has a similar effect (Balan and Verestiuc, 2014; Konieczynska et al., 2016; Zhao et al., 2017; Zou et al., 2022). However, further improvements in adhesion properties are difficult to achieve by relying on these materials alone, as the presence of a hydrated film can strongly interfere with the adhesion of the material. Introducing hydrophobic groups to disrupt the hydration film and allow better contact between the adhesion groups in the material and the tissue surface is an effective solution (Pinnaratip et al., 2019). Inspired by barnacle cement proteins, Ni created a series of dynamic phenyl borate ester-based adhesive hydrogels by coupling cation-structured polyphosphazene with polyvinyl alcohol (PPBA-PVA) (Ni et al., 2022). In a liquid environment, hydrophobic aromatic groups disrupt the hydration film, and the hydrogel achieves an adhesive strength of 45 kPa through cation-π and π-π interactions and hydrogen bonding.

The incorporation of nanocomponents with a porous structure and a high specific surface area can help increase the adhesion properties of materials (Hu et al., 2022). Tian et al. (2022) incorporated mesoporous bioactive glass nanoparticles (MBGNs) into a material system to make DGM and increased the adhesive strength to 107.55 kPa by increasing the cohesion of DGM (Tian et al., 2022). Due to the high temperature sensitivity of these substances, painless removal of the material can be performed by changing the ambient temperature (e.g., by introducing ice). The diatom biosilica, which contains a large number of surface silanol groups, was incorporated into bletilla striata polysaccharide, a traditional Chinese medicine ingredient with haemostatic effects (Sun et al., 2023). The structural features of the diatom biosilica increased the cohesion of the material and the force applied to the tissue surface to improve the contact efficiency and duration of the material with the wound, reducing the bleeding volume from 452 mg to 250 mg and the bleeding time from 327 s to 186 s. Diatom biosilica was also used to reduce the bleeding time from 327 s to 186 s.

### 4.2 Enrichment of blood cells

Primary thrombus formation is accomplished by the rapid aggregation and activation of red blood cells and platelets. Therefore, the design of many haemostatic materials focuses on the adsorption and concentration enhancement of red blood cells and platelets (Zhu et al., 2018). The longest used strategy is to provide adhesion sites for blood cells as well as to absorb water from the blood. Many substances with this function exist in nature. Chitosan, gelatine and their derivatives are the most common raw materials for haemostatic materials.

Chitosan is a positively charged polysaccharide that can adsorb blood cells and clotting factors through electrostatic interactions to achieve haemostasis (Chou et al., 2003; Okamoto et al., 2003; Lestari et al., 2020). Chitosan has excellent biocompatibility, biodegradability, and low toxicity, undergoes enzyme-controlled degradation, and contains easily modified free amino and hydroxyl groups (Kean and Thanou, 2010). Therefore, it is widely used in various kinds of haemostatic hydrogels. Feng and Wang (2022) mixed chitosan with graphene oxide to further enhance the ability of the material to enrich blood cells through the strong adsorption of graphene oxide. In rat liver injury experiments, the bleeding time was reduced by more than 60% and the bleeding volume by more than 75% compared to that in the control group. However, the water solubility of chitosan is not ideal. Many researchers have chosen to use CMCSs as a raw material for further synthesis. Not only does CMCSs have better water solubility, but the addition of carboxyl groups also increases the material’s adsorption of blood cells (Huang et al., 2016). Rao et al. (2022) Combined CMCSs with TA-stabilized silver nanoparticles in a haemostatic gel that can be used for haemostasis in minimally invasive surgery. Poly-gamma-glutamic acid (γ-PGA) can expel water from the wound surface, which can rapidly concentrate blood to increase the concentration of blood cells at the wound site, and combining this property with the adsorption effect of CMCS can achieve a significant reduction in bleeding time (Chen et al., 2023). ε-PL and 1,4-benzenediboronic acid (BDBA) can play a role in regulating the crosslinking strength and gelation rate of hydrogels, and haemostatic gels with different pore structures and gelation times were prepared with different degrees of crosslinking. The most suitable preparation schemes were screened through various application scenarios. The extent of bleeding in mouse livers treated with the hydrogels could be reduced from 240 to 55 mg (77% reduction) (Wang YC. et al., 2020; Geng et al., 2020).

Hydrophobically modified chitosan (hmCS) with hydrophobic aliphatic side chains has excellent erythropoietic capacity (Dowling et al., 2011; Dowling et al., 2015; Duc-Thang and Lee, 2017). Hydrogels prepared from hmCS with ODex using the Schiff base reaction can effectively reduce the clotting time of heparinized WB (Du et al., 2019b). Kong et al. (2021) prepared a series of CuS NP hydrogels using the derivative N-carboxyethyl chitosan (CEC), sodium oxide alginate (OA) with good water absorption, and CuS nanoparticles with high specific surface positivity, which effectively increased the enrichment efficiency of haemocytes. In an *in vivo* study in a rat liver bleeding model, the blood loss was 185.1 ± 18.6 mg in the CEC−OA2.8−CuS0.8 group and 538.6 ± 27.5 mg in the control group (Kong et al., 2021). Choline phosphoryl (CP) is the reverse orientation of phosphatidylcholine (PC, the head group of phospholipids) and can adsorb to adsorbed biofilms by electrostatic interactions. Choline-phosphorylated functionalized chitosan (CS-g-CP) was prepared by grafting CP onto CS and mixing it with OD to obtain self-repairing haemostatic hydrogels (CS-g-CP/ODex). The haemostatic capacity of CS-g-CP/ODex was significantly greater than that of commercially available fibrin sealant in the tail amputation, liver, and spleen of rats (Zhu et al., 2022). Wang prepared with the aid of UV crosslinking CS/4-PA/CAT from dihydrocaffeic acid (CAT), 4-glutamic acid, and chitosan to form a haemostatic hydrogel with a hydrophobic network, which overcame the problem of the material dissolving and extruding the periwound tissue (Wang et al., 2023). Grafting gallic acid (GA) onto CS increased the mechanical strength of the material. Gong et al. (2023) incorporated iron oxide nanoparticle-loaded mica nanosheets with a typical 2D lamellar morphology and negatively charged surface (iron oxide nanoparticles@mica, IM) with a typical 2D sheet-like morphology and negatively charged surface to make a composite magnetic hydrogel, which was able to reduce the bleeding time in a rat model of liver injury by 60% (Yan et al., 2022; Gong et al., 2023).

Gelatine is a hydrolysed product of collagen that retains similar biological functions and has good water absorption and blood cell adhesion properties (Zeugolis et al., 2008; Xu et al., 2013; Yang et al., 2018). However, the mechanical strength of these materials is poor, and they need to be modified or combined with other components. In early research, gelatine and SA were combined to make a precursor gel, which was first used to cover the wound surface and subsequently crosslinked *in situ* using a spraying portion composed of TA and Ca^2+^ chloride to form the low-swelling haemostatic hydrogel A_G_B_S_ (Wang et al., 2022). Hydrogels made from gelatine and hyaluronic acid have also been shown to have haemostatic properties superior to those of fibrin glue. In a rat bleeding model, the fibrin glue group had a blood loss of 109 ± 92 mg, whereas the blood loss in the G/HA hydrogel group was 64 ± 50 mg (Luo et al., 2020). Replacing gelatine with gelatine methacryloyl (GelMA) allows the introduction of a photocrosslinked network into the material system and increases the stability of the hydrogel’s three-dimensional network structure. It is often used to construct the base skeleton for loading other haemostatic components (Zhang et al., 2022; Haghniaz et al., 2023). The incorporation of silicate nanosheets (SNs) into GelMA-based composite hydrogels was shown to reduce bleeding in rat liver injury by 50% (Gaharwar et al., 2014).

In addition to gelatine, a number of other peptides or protein derivatives with RGD sequences also have favourable blood cell adhesion effects (Quan et al., 2016). Zhai et al. (2019) designed a haemostatic hydrogel with encouraging haemostatic properties via the coassembly of cell adhesion peptide concatenate (Pept-1) and alginate (ALG) and reduced the amount of bleeding to 18% of the untreated control in a liver puncture mouse model (Zhai et al., 2019). Pept-1/ALG utilizes the RGD fraction present in Pept-1 to bind to the a8b3 receptor on platelets and accelerate haemostasis. Notably, ADP is a platelet-activating factor that readily self-assembles with other peptides. ADP adsorbs platelets and activates platelets, causing the activated platelets to release biologic factors and further enriching platelets to promote coagulation (Lokhande et al., 2018). Zhou et al. (2022) prepared a J-1-ADP hydrogel by self-assembly of the antimicrobial peptide jelloprotein-1 (J-1) and ADP. In a rat liver bleeding model, the blood loss in the J-1-ADP hydrogel treatment group was 41.15 ± 14.43 mg, while the blood loss in the no treatment and PBS treatment groups was 178.08 ± 41.53 and 167.03 ± 23.36 mg, respectively (Zhou et al., 2022).

Some surface-charged materials, such as graphene oxide, limonite nanoclay, and metal nanosheets, can adsorb blood cells through electrostatic interactions to achieve rapid thrombus formation (Gaharwar et al., 2014; Gaharwar et al., 2019; Gu et al., 2023). The addition of such substances not only increases the adsorption of the material to blood cells but also improves the cohesion of the material and increases the mechanical strength. Wang designed a bionic self-assembling peptide (BSAP) hydrogel that is extremely sensitive to Ca^2+^ and coagulation factor XIIIa and can rapidly adsorb and activate platelets and erythrocytes upon contact (Wang et al., 2022).

### 4.3 Activation of the coagulation cascade

Haemostasis must ultimately rely on the activation of the coagulation cascade, which can be effectively achieved by the direct introduction of coagulation factors into the body. Ca^2+^, also known as coagulation factor IV, is an important part of the coagulation cascade. It has become one of the commonly used haemostatic components due to its low cost, easy availability and adjustable mechanical strength. It has been demonstrated that excellent haemostatic effects can be obtained by adding Ca^2+^ directly to the material system without special treatment (Sundaram et al., 2021; Wang et al., 2021). In Weng’s study, the Ca^2+^-containing hydrogel NSC-OHA showed blood loss similar to that of a commercial haemostatic agent (Arista™) in an *in vivo* experiment in rats (0.08 ± 0.02 g, 0.10 ± 0.03 g), both of which were significantly lower than that of the control group (0.62 ± 0.08 g), demonstrating the excellent haemostatic effect of NSC-OHA. Pillai found that the addition of Whitlockite nanoparticle nWH, which can release Ca^2+^, to the haemostatic material was able to reduce the blood loss of rat liver perforation by 50% (Pillai et al., 2019).

Thrombin plays an important role in the whole coagulation process and can activate the conversion of fibrinogen to fibrin. Platelet-rich plasma (PRP) activated by thrombin is combined with sodium alginate-Fe^3+^ to prepare a hydrogel with a dual network structure that prevents excessively rapid release of the loaded components and ensures sufficient coagulation components in the patient’s body (Wang et al., 2023). Applying the material to the wound surface is equivalent to placing a blood clot at the wound site, fast-tracking the haemostatic process. However, these “artificial thrombi” lack sufficient tissue adhesion properties. Xu et al. (2023) used 6-aminobenzo [c] [1,2] oxaborol-1 (3H)-o (ABO)-conjugated hyaluronic acid (HA-ABO) and DA-grafted alginate (Alg-DA) as the backbone components of the hydrogel (Xu et al., 2023). The excellent adhesion properties and self-healing properties of the materials reduced blood loss from 364.1 ± 52.4 mg to 129.4 ± 23.0 mg. Transglutaminase (factor XIIIa) promotes fibrin network formation and increases thrombus strength. Glutaminase can not only be converted to transglutaminase in the haemorrhagic environment but also be used as a crosslinking agent to prepare hydrogels (Zhou et al., 2019). Sun OPEG-AG-G hydrogels were prepared via double-tube injections (Sun et al., 2022). Tube A contained 10% glutamine and 20% aminated gelatine, and tube B contained 10% oxidized PEG and 1% transglutaminase. OPEG-AG-G achieved haemostasis of porcine arterial rupture without vascular closure.

Activators of distinct coagulation pathways can be coincorporated into the material system to improve haemostasis. Sundaram et al. (2019) found that incorporating nanobio-glass (nBG) containing silica (which activates coagulation factor XII), Ca^2+^ (which activates the endogenous system), and phosphate ions (which initiate the exogenous pathway) into chitosan matrix hydrogels can promote rapid thrombus formation (Sundaram et al., 2019). Loading RG5, which promotes the coagulation cascade reaction, into platelet-activating hydroxyapatite nanotubes (HNTs) to make a haemostatic powder, which is subsequently loaded into an ALG and gelatine hydrogel skeleton, was the goal of Zhang. The prepared HNTs/RG5 possessed haemostatic properties superior to those of commercially available haemostatic products (the haemostasis times of the HNTs/RG5-and Celox®-treated groups were 97.8 ± 8.2 s and 136 ± 22.1 s, respectively) (Zhang TW. et al., 2021). The strategy of using a combination of hydrogel and haemostatic powder results in the superior performance of AGHR hydrogels, which remain injectable even in extremely hot and cold regions and are suitable for many types of first aid and battlefield rescue.

## 5 Summary and outlook

Haemostasis is a key challenge in post-trauma treatment. Although the body’s inherent coagulation mechanism can complete the haemostatic repair of small-scale wounds through vasoconstriction, platelet plug formation and coagulation cascade triggering, rapid haemostasis cannot be naturally achieved in large-area trauma bleeding, bleeding from important vessels (such as the common carotid artery and femoral artery), and bleeding from parenchymal organs (such as the liver and heart) solely through the coagulation mechanism alone, and additional injury or even death can ensue. Here, we summarize the main haemostatic methods, including haemostatic compression, which is effective at stimulating vasoconstriction and reducing blood flow but may cause limb necrosis. These methods are currently used only in first aid and require the next step of treatment as soon as possible. Haemostatic materials with strong water absorption and blood cell adsorption capacity can rapidly increase blood cell concentrations and create a high clotting environment, and gelatine sponges are widely used in intraoperative and postoperative haemostasis. These materials are completely dependent on the patient’s coagulation system and are not suitable for patients with coagulation disorders, and some highly absorbent materials may cause local tissue water loss. Thrombinogen and fibrinogen components can effectively promote coagulation cascade reactions and accelerate the formation of blood clots, while the direct addition of coagulation components can help patients with coagulation disorders quickly stop bleeding. However, these materials present problems such as virus transmission, scarce sources, and high cost. Materials with high adhesion properties can bind wounds to achieve haemostatic effects. At present, relatively few haemostatic adhesives, mainly cyanoacrylate and fibrin sealants, are commercially available. Cyanoacrylate adhesives have strong adhesive properties and fast haemostatic efficacy, but they may produce formaldehyde and other harmful degradation products during biodegradation, which need to be considered when using these materials. Fibrin sealants achieve haemostasis through the activation of fibrinogen and thrombin *in vivo*, but they may allow viral transmission and are expensive, limiting their use in clinical practice. Therefore, further research is needed to develop safe, effective, and cost-saving haemostatic adhesives for wound closure.

As mentioned above, a large number of haemostatic products have been used in clinical practice. However, there are still many unresolved issues. One of the most difficult problems is noncompressible haemorrhage, in which direct access to the bleeding site is difficult and bleeding cannot be directly controlled by physical pressure, making this type of injury difficult to address. Clinical treatment is still based on blood transfusion and surgery, but this approach is difficult to implement in emergencies and in war environments. Hydrogels have the advantages of high hydrophilicity, high biosafety, easy modification, easy loading, etc., and are among the most promising materials. Hydrogels with injectable properties are beneficial for haemostasis in incompressible haemorrhage, in narrow and deep wounds, and in minimally invasive surgeries. Researchers have prepared injectable haemostatic hydrogels in a variety of ways. Studies have confirmed that positively charged raw materials or groups, aldehyde structures and catechol structures have positive effects on the adhesion strength of materials and contribute to the rapid closure of wounds. Haemostatic materials that can promote blood cell enrichment can be prepared by selecting materials that have the characteristics of electrostatic adsorption by blood cells and rapid water absorption. Loading coagulation factors, haemostatic peptides and other procoagulant substances can accelerate the progression of the coagulation cascade, which is highly important for patients with coagulation disorders. Notably, the three-dimensional mesh structure of hydrogels easily loads drugs and bioactive factors, which contributes to the expansion of hydrogel functions. Amoxicillin (Liang et al., 2019b; Qu et al., 2019), ampicillin (Refat et al., 2018), tetracycline (Anjum et al., 2016; Rakhshaei and Namazi, 2017; Liu et al., 2018), gentamicin (Singh et al., 2013; Zhang et al., 2015), ciprofloxacin (Radhakumary et al., 2011; Shi et al., 2015; Gao et al., 2019), moxifloxacin (Singh and Dhiman, 2015; Singh et al., 2016), chloramphenicol (Lacin, 2014), sulfadiazine (McMahon et al., 2016), simvastatin (Rezvanian et al., 2017), salicylate (Mi et al., 2011), and other components have been encapsulated in hydrogels to confer antibacterial properties. Some metal ions, such as silver, gold, and zinc, can also have antibacterial effects, and their complexation with the main structure of the hydrogel can also increase the strength of the system (GhavamiNejad et al., 2016; Chen et al., 2019; Liang et al., 2019c; Mahmoud et al., 2019; Shi et al., 2019; Tang et al., 2019). Stem cells can secrete essential factors that facilitate wound repair and stimulate angiogenesis and re-epithelialization. Therefore, many hydrogels promote wound repair by loading stem cells (Oryan et al., 2019; Xu et al., 2019; Yao et al., 2019; Xiong et al., 2022).

Although injectable hydrogels have achieved rapid haemostasis with high adhesion, high water absorption, enrichment of blood cells, and facilitation of the coagulation cascade in past studies, there are still many challenges to overcome. First, the speed and manner of *in situ* hydrogel formation should be further optimized. To better adapt to the wound shape and achieve injectable properties, many hydrogels are prepared by *in situ* gelation: a syringe is used to inject the precursor solution at the wound site and Schiff base crosslinking and photoccrosslinking occur simultaneously to achieve *in situ* gelation at the wound site. Although, after continuous optimization and improvement, many gels can now form within 30 s, there is still a risk that such a gelling speed on the wound surface is insufficient in cases of severe traumatic bleeding or vascular bleeding; the need for UV light and other light sources to achieve gelling also limits the use of gels and increases biosafety risk. Second, exisiting research has mostly used medium- and small-animal models, such as a rat liver injury model, a rat femoral artery injury model, a rat broken tail model, a rabbit ear marginal artery injury model, and a rabbit liver injury model for *in vivo* experimental verification. Although the haemostatic performance of the material can be verified to a certain extent, limited by the animal’s size, blood volume, bleeding intensity and other issues, there is still a large gap in understanding the actual clinical bleeding situation. It is recommended that animal models more closely related to the clinical situation should be created in future studies or that large animal models be used for further validation at a more mature stage of material research. Third, implantable biomedical materials will stay in the body for a long time. However, the existing studies mostly focus on short-term haemostatic performance evaluation or simple *in vitro* safety evaluation (such as red blood cell haemolysis tests and *in vitro* safety tests), lacking long-term biocompatibility assessment and posthemostasis prognosis evaluation. These are the key factors determining whether materials can be put into clinical use. In the history of haemostatic material application, there are many examples of excellent haemostatic performance but secondary damage to patients, such as endothelial damage and systemic thrombosis caused by WoundStat^®^ and tissue inflammation and tissue sclerosis caused by the degradation of cyanoacrylate products. Our group also found in previous research that haemostatic hydrogels with strong adhesion properties can cause aggravated tissue adhesion, which is one of the complications that need to be prevented after traumatic bleeding. All these problems can be addressed and further ameliorated through perfect and systematic long-term safety experiments. We hope that researchers can give close attention to long-term safety investigations in future work. Finally, in situations such as car accidents, natural disasters, and war, there are often multiple types of trauma, and there is no visual identification of the bleeding site. Existing injectable hydrogel haemostatic materials require a clear view of the treatment and direct contact with the wound, and there is a gap in addressing actual clinical needs. In addition to injectable hydrogels, targeted drugs can be developed for intravenous injection in combination with related devices or intelligent equipment developed for rapid diagnosis, accurate positioning, and stable haemostatic integration intelligent haemostatic robots. These new technologies and devices have the potential to address the challenges of multiple and diffuse trauma bleeding events and provide more convenient and efficient treatment options for trauma patients.

The development of perfect haemostatic injectable hydrogels is still a long way off, but we hope that this paper will help researchers interested in understanding the current state of injectable haemostatic hydrogels and has provided some inspiration for the research efforts of those working to advance this field. We encourage further research and innovation in this area to address the remaining challenges and to improve the treatment options available for trauma victims.
